# actifpTM: a refined confidence metric of AlphaFold2 predictions involving flexible regions

**DOI:** 10.1093/bioinformatics/btaf107

**Published:** 2025-03-13

**Authors:** Julia K Varga, Sergey Ovchinnikov, Ora Schueler-Furman

**Affiliations:** Department of Microbiology and Molecular Genetics, Institute for Biomedical Research Israel-Canada, Faculty of Medicine, The Hebrew University of Jerusalem, Jerusalem 9112001, Israel; Department of Biology, Massachusetts Institute of Technology, Cambridge, MA 02139, United States; Department of Microbiology and Molecular Genetics, Institute for Biomedical Research Israel-Canada, Faculty of Medicine, The Hebrew University of Jerusalem, Jerusalem 9112001, Israel

## Abstract

**Summary:**

One of the main advantages of deep learning models of protein structure, such as Alphafold2, is their ability to accurately estimate the confidence of a generated structural model, which allows us to focus on highly confident predictions. The ipTM score provides a confidence estimate of interchain contacts in protein–protein interactions. However, interactions, in particular motif-mediated interactions, often also contain regions that remain flexible upon binding. These noninteracting flanking regions are assigned low confidence values and will affect ipTM, as it considers all interchain residue–residue pairs, and two models of the same motif-domain interaction, but differing in the length of their flanking regions, would be assigned very different values. Here, we propose actual interface pTM (actifpTM), a modified ipTM measure, that focuses on the residues participating in the interaction, resulting in a more robust measure of interaction confidence. Besides, actifpTM is calculated both for the full complex as well as for each pair of chains, making it well-suited for evaluating multi-chain complexes with a particularly critical binding interface, such as antibody-antigen interactions.

**Availability and implementation:**

The method is available as part of the ColabFold (https://github.com/sokrypton/ColabFold) repository, installable both locally or usable with Colab notebook.

## 1 Introduction

Since 2020, AlphaFold2 [AF2 (Evans *et al.* 2021, Jumper *et al.* 2021)] and other similar deep learning algorithms (Baek *et al.* 2023, Ahdritz *et al.* 2024) have revolutionized structural biology, by making swift and confident predictions of millions of protein structures and complexes available. Their appearance was also groundbreaking in terms of scoring of generated models, as they were trained to be able to assess the confidence of the predictions, along with the output. One of the scores introduced in AF2-Multimer that is used for assessing complex confidence is ipTM (interface predicted template modeling score), which evaluates the predicted relative confidence of binding partners (Evans *et al.* 2021, Jumper *et al.* 2021).

**Figure 1.**
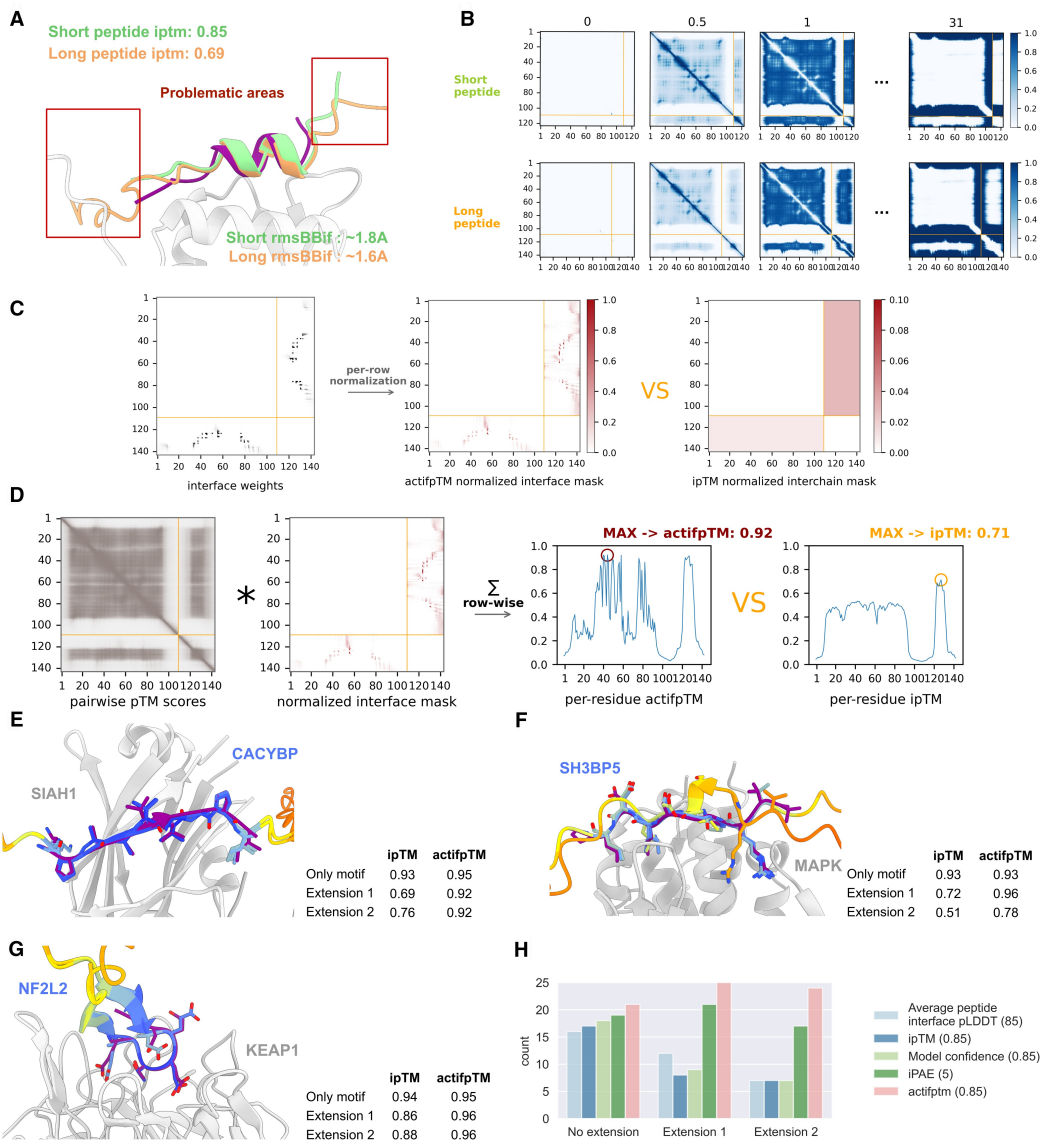
actifpTM in the AF2 pipeline helps correcting bias from flexible flanking regions. (A) Predictions of MDM2-p53 with p53 peptides of length 14 (15–29) and 34 (5–28) [green and orange, respectively; the native peptide is colored in dark magenta, PDB ID: 1YCR (Kussie *et al.* 1996)] demonstrate the decrease in ipTM for the longer peptide, even though it is slightly more accurate than the short one (rmsBB_if: RMSD across peptide interface backbone atoms). (B) The predicted error matrices for each error bin (the first three and the last error bins are shown out of 64) are very similar for the short and long peptide predictions, except for the high error for the flanking regions present only in the longer peptide (white regions). This does not support the large drop in ipTM value upon elongation of the peptide. (C) In the last steps, the pairwise pTM-score matrix (see Supplementary Fig. S1 for calculation) is multiplied by the actifpTM normalized residue weights, then summed for every residue (row-wise). (D) As with the original TM-score, the maximum of the values along the sequence is selected as the final actifpTM score. (E–G) Comparison of ipTM and actifpTM values for example systems. The peptides were predicted with three different lengths: only the motif region, adding flanking regions of same length as the motif, or adding flanking regions twice the motif length (Lee *et al.* 2024). On all panels, dark magenta denotes experimentally resolved peptides, and modeled peptides are colored according to AF2 pLDDT. (E) When the modeled conformation of the defined region of the peptide does not change, actifpTM provides very similar scores, while ipTM demonstrates a considerable decrease [SIAH1 & CACYBP, PDB ID: 2A25 (Santelli *et al.* 2005)]. (F) actifpTM provides a similar score for similar predictions but drops appropriately upon less confident predictions [MAPK & SH3BP5, PDB ID: 4H3B (Laughlin *et al.* 2012)]. (G) actifpTM tracks slight changes in conformation more appropriately than ipTM, by not taking into account residues outside the interface. Upon extensions, the peptide prediction became more confident by introducing a beta-hairpin structure in the flanking regions. Unlike actifpTM, ipTM completely misses the overall increase in prediction confidence [KEAP1 & NF2L2, PDB ID: 3ZGC (Hörer *et al.* 2013)]. (H) With a strict threshold (indicated in parentheses), actifpTM has a higher acceptance rate than the other metrics that can be calculated from AF2 confidence outputs, in particular for predictions that include flanking regions of the peptide. For all structure visualization ChimeraX v1.8 (Meng *et al.* 2023) was used.

Peptide–protein interactions are widespread in cells and involved in many important regulatory processes (Babu *et al.* 2011). Although modeling interactions between proteins is generally challenging, modeling of peptide–protein interactions has to address one major additional challenge, namely the flexibility of the peptide partner and the fact that we usually do not know or have an estimate of their free, unbound structure, unlike for domain-domain interactions (Ciemny *et al.* 2018). This complicates the search for the native bound structure, significantly increasing computation time. Additionally, the bound conformations can still be somewhat flexible, especially the flanking regions surrounding a more tightly bound region (Bugge *et al.* 2020). ipTM (and a confidence metric, defined as a weighted sum of ipTM and pTM) is a frequently used score for assessing docked peptide–protein models (Teufel *et al.* 2023, Bret *et al.* 2024, Lee *et al.* 2024), but it was trained on PDB structures without long intrinsically disordered regions or flexible flanking regions of peptides, since these are usually not resolved in crystal structures. Although it is generally referred to as interface pTM, it is actually defined as an inter*chain* pTM score, as it takes into account all residue–residue pairs across all chains of a complex with equal weight, regardless if they are actually part of the interface or not. Therefore, the score changes significantly if a sequence that includes also nonstructured flanking regions is provided (e.g. by including disordered regions to extend a defined domain or peptide motif, see Fig. 1A), making comparison between otherwise similarly well modeled complexes challenging. This phenomenon particularly affects peptide–protein-like interactions as their binding interface regions are very short.

A seemingly obvious solution to the problem would be to run predictions on minimal binding regions without the flanking sequences. This has however been shown to be a less effective method for modeling (Bret *et al.* 2024, Lee *et al.* 2024). Even though these regions are usually flexible and not necessarily confidently modeled, AF2 seems to utilize the context for making accurate predictions. Alternatively, one can drop regions based on other confidence metrics and calculate the interface predicted alignment error score (ipAE) (Kim *et al.* 2024, Kruse *et al.* 2024) for the confident interface residues. However, since ipTM is more sensitive than ipAE (Teufel *et al.* 2023, Bret *et al.* 2024, Lee *et al.* 2024), it would be advantageous to modify ipTM directly (and consequently, the confidence score). In this study, we introduce actual interface pTM (actifpTM), a modified ipTM score that focuses on confident interface residues. To do so, we first provide an example that highlights the need for this new score. We then detail how the original ipTM is calculated, and describe how this calculation was modified for actifpTM to account for the relevant residues only.

## 2 Results

Figure 1A shows a representative result of an AF2 prediction of a peptide-receptor interaction: structural models of p53 binding to MDM2. The prediction was performed with two different lengths of the p53 peptide (short: 15–29, long: 5–28). Even though the prediction is of comparable accuracy for the binding motif (both around 1.7 Å RMSD of the peptide interface residue backbone atoms), and pLDDT confidence predictions are similar (see coloring scheme), ipTM values differ dramatically. This phenomenon is a recurring issue when assessing peptide–protein models.

### 2.1 Implementation of the modified score

This motivated us to implement a score which is not biased by such flexible flanking regions, but behaves similarly to ipTM, as it was already proven to be excellent for scoring complexes without such disordered regions. For better understanding of our modifications, here we briefly explain how the ipTM score in AF2 is calculated based on the equation of the original TM-score (Zhang and Skolnick 2004) (see Supplementary Fig. S1):


$${TM}_{\mathrm{score}}=\max\left[\frac{1}{L_{\mathrm{target}}}\sum_{i}^{L_{\mathrm{common}}}\frac{1}{1+{\left(\frac{d_{i}}{d_{0}\left(L_{\mathrm{target}}\right)}\right)}^{2}}\right]$$

which sums contributions from each pair of aligned residues (with length of the alignment *L*_common_, target length *L*_target_, normalization *d*_0_, and residue distance *d_i_*; the max over all possible alignments of the structures is taken. Note that the pTM-score in AF2 uses a modified approach where the *L*_common_ = *L*_target_ = total length of the concatenated input).

The error head produces a 3D matrix of probabilities for each of 64 distance bins (0–0.5, 0.5–1, …, 30.5–31 Å, Fig. 1B). A maximum theoretical TM-score is computed for each bin using its midpoint as *d_i_*, and these scores—weighted by their bin probabilities—are summed into a 2D pairwise pTM-score matrix (Supplementary Fig. S1A). For pTM, this entire matrix is used in the next step; for ipTM, intrachain residue–residue pairs are masked with zeros. The resulting matrix is row-wise normalized and multiplied by the residue mask (Supplementary Fig. S1B). Subsequently, each row is summed (Supplementary Fig. S1C). The maximum of these per-residue sums is the final ipTM value, effectively emulating all possible residue alignments and reporting the best TM-score. In the original AF2 publication, this is considered to be equivalent to the model and the native structure being superimposed onto each other on every possible residue and reporting the TM-score of the best alignment (Evans *et al.* 2021, Jumper *et al.* 2021).

The above-described calculations assign equal weights to each residue pair, d_i_, regardless of them being in contact with each other or not. To overcome this issue, for actifpTM we modify the masking of the original ipTM calculations to take into account the predicted distance probabilities as residue-pair weights (de facto, skipping d_i_ values of noninteracting residues, Fig. 1C and D). Consequently, the score does not depend anymore on the length of the flanking regions, making it close to uniform across predictions.

The main difference between calculating ipTM and actifpTM is the residue weights used in the calculations (Fig. 1C, left versus right). Originally, residue weights are set to 1 for the whole interchain areas, then normalized across rows (Supplementary Fig. S1B). Instead, we used the contact probability map output by AF2 and calculated the probabilities of each residue-pair to be within 8Å distance to each other (Cβ-Cβ atoms, Cɑ is taken for glycines) (Fig. 1C). Reassuringly, the resulting pseudo-contact map of the confident prediction is in good agreement with the contact map derived from the solved structures (Supplementary Fig. S2). Next, we follow the usual pTM-score calculation: the residue-weights are row-wise normalized (Fig. 1D, center), and the theoretical maximum TM-scores for each error bin are calculated, similarly to the original TM-score (Supplementary Fig. S1C). These values are multiplied by their respective error matrices and summed for each row. The maximum of these per-residue actifpTM scores is taken as the final actifpTM score (Fig. 1D).

The calculation of actifpTM required modifying the AF2 source code and was integrated into the widely used ColabFold (hence also localcolabfold) pipeline (Mirdita *et al.* 2022), and can be invoked by checking the calc_extra_ptm checkbox in the Jupyter notebook when running on Colab (Supplementary Fig. S3A), or by using the --calc_extra_ptm flag when running localcolabfold. Unlike the one ipTM value calculated across all chains that is provided in the original implementation, we provide in addition actifpTM values for every pair of chains. In the implementation, the pairwise ipTM and per-chain pTM values are also reported and plotted together with the pairwise actifpTM (Supplementary Fig. S3B–D).

### 2.2 actifpTM—case-studies and overall assessment

The implemented code was run on the dataset from Lee *et al.* (2024) in which problems in estimating ipTM arising from flanking regions were detected earlier, and results for the minimal binding motif were compared to those including extensions of two different lengths: (i) extension 1 — short flanking sequence: extension with the length of the motif added on both sides, or (ii) extension 2 — long flanking sequence: extension with twice the length of the motif added on both sides. Both ipTM and actifpTM were calculated for all predictions. In the following, we describe a few examples that highlight the advantage of the new actifpTM measure. For clear comparison, we select the AF2 model that provides the best ipTM for the prediction without extension (e.g. #3) and use this same model for all runs (*i.e.* #3, for the predictions without, with short and long flanking sequence extensions).

1. Peptide of CACYBP bound to SIAH1 complementing its beta-sheet with a short beta-strand (Fig. 1E): The predictions for the two different types of extensions are very similar (also in terms of pLDDT of the peptide region resolved in the crystal structure). However, the ipTM value for the model using the longer peptide extension is dramatically reduced from 0.93 to 0.69. The actifpTM decreases slightly, from 0.95 to 0.92, but this is negligible, and overall still allows for much more accurate assessment of the model than ipTM.
2. Binding of SH3BP5 peptide with an extended motif to the binding site of MAPK (Fig. 1F): there is a considerable change in the binding conformation upon a long extension of the flanking regions (Fig. 1F, extension 2), although the peptide is still located in the binding site. This change in conformation and also in confidence is reflected in the value of actifpTM, dropping from 0.93 to 0.78. The slight increase between the prediction with only the motif and extension 1 is not surprising since it has already been shown that adding flanking regions to predictions often increases model accuracy (Bret *et al.* 2024, Lee *et al.* 2024).
3. Beta hairpin formation in the interaction between the Kelch domain of KEAP1 and NF2L2 (Fig. 1G): although the minimal version of the motif is already very confident (ipTM and actifpTM 0.94 and 0.95, respectively), extending allows for forming a beta-hairpin. The actifpTM increases very slightly on this change of conformation, which usually makes binding tighter by decreasing the entropy of the peptide, while ipTM drops, as a result of the ends of the flanking regions not confidently modeled.

Analysis of the results for the full dataset reveals that actifpTM provides highest success rate among different measures (Fig. 1H, where success is defined as average peptide interface pLDDT >85; ipAE <5.0Å [median of PAE values across interface residues within 8Å (Teufel *et al.* 2023)]; ipTM >0.85; and model confidence >0.85 [0.8*ipTM + 0.2*pTM]). As expected, the difference is most pronounced for models that include flanking regions [one or two extensions (Bret *et al.* 2024, Lee *et al.* 2024)]. In these cases, ipAE and actifpTM indeed perform best, with actifpTM outperforming ipAE, in agreement with previous studies that have reported better performance for ipTM compared to ipAE (Teufel *et al.* 2023, Bret *et al.* 2024, Lee *et al.* 2024).

## 3 Discussion

This study implements actifpTM, a modified version of the AF2 ipTM score, to account for the flexible context of the binding region of peptides. As per its original implementation, ipTM suffers from a bias introduced by these regions, since it considers full chains instead of the interface between binding partners. We implemented a simple way to overcome this limitation and modify the score by weighing interactions between two chains based on their confidence. We demonstrate the merit of actifpTM across several examples and have incorporated it into widely used versions of AF2.

Note that this method only produces equal scores when predictions have similar confidence levels; it cannot boost an overall low-confidence prediction to a high-confidence one. However, ipTM has been shown to be good at distinguishing between accurate and inaccurate predictions, sometimes even separating binding from nonbinding peptides (Teufel *et al.* 2023). By correcting for unbound shorter or longer disordered regions around the binding residues, we rendered this confidence metric less biased and comparable across predictions of different complexes, which is especially useful for large-scale screening approaches.

Previous studies have also approached the challenge of flexible flanking regions, by e.g. considering only confident positions when calculating ipAE (Kruse *et al*. 2024; Kim *et al*. 2024), we note that for the definition of confident interface residues an additional cutoff (namely which residue pairs are dropped from evaluation of the interface) needed to be defined (Kruse *et al*. 2024; Kim *et al*. 2024). Finally, several studies have shown that ipTM was better than ipAE at selecting models (Teufel *et al.* 2023; Lee *et al*. 2024; Bret *et al*. 2024).

Following our work, a recent method dealing with the same problem proposed the use of the PAE matrix to calculate a pTM-like metric, called ipSAE (Dunbrack 2025). In the preprint, the method was compared to actifpTM on six different complexes. In three out of six complexes, the actifpTM value identifies correctly high confidence, well-docked peptides (actifpTM > 0.9, full-atom RMSD < 2Å). In two cases, the interaction is not confidently modeled, which actifpTM also correctly denotes (actifpTM < 0.5). In the case of the MAPK10 and full-length SH3BP5, the two metrics provide different results (actifpTM = 0.69, ipSAE = 0, as a different region is put into the binding site). Since the actifpTM value for this prediction is below the confidence threshold (it is derived from ipTM, often calibrated to be reliable above 0.8 or 0.85), we manually inspected this prediction. In fact, SLiMScan (https://slim.icr.ac.uk/tools/slimscan/) identifies it as a possible NEK2 phosphorylation motif, based on the MOMAP database (https://slim.icr.ac.uk/momap/). Without further experiments, we cannot conclude whether this prediction is indeed an AF2 artifact, since a possible (weak) interaction cannot be ruled out. The corresponding actifpTM value reflects this ambiguity. We anticipate that actifpTM will also be useful to identify additional similar borderline cases that can be further studied to refine predictive as well as experimental assessment of the relevance of SLIM-receptor interactions.

We expect that this scoring approach will be particularly useful for assessing predictions of peptide–protein interactions, as the penalty of disordered regions around the binding site is the highest in these cases due to the small number of binding residues. Moreover, actifpTM (as well as the original ipTM and chain pTM) is now implemented and displayed not only for a full complex, but also for each pair of chain separately (Supplementary Fig. S3B and C). This is especially beneficial for accurately assessing pairwise chain interactions within multi-chain complexes, by restricting the evaluation to interfaces of interest. A good example for the latter is the case of antibody-antigen complexes, where the focus of interest is usually on the interaction of the antibody with the antigen, whereas the interaction between the heavy and light chains is usually predicted with high confidence.

## Supplementary Material

btaf107_Supplementary_Data

## Data Availability

The method is available as part of the ColabFold (Mirdita *et al.* 2022) (https://github.com/sokrypton/ColabFold) repository, by checking the *calc_extra_ptm* checkbox in the AlphaFold2.ipynb Jupyter notebook when running on Colab, or by using the --*calc_extra_ptm* flag when running localcolabfold. A Colab notebook to reproduce figures in this paper is available on GitHub (the notebook was run on a T4 GPU, using Python 3.10.12 and CUDA v12.2): https://doi.org/10.5281/zenodo.14515382.

## Declaration of usage of generative AI and AI-assisted technologies

During the preparation of this work, the authors used ChatGPT to improve language and readability and ChatGPT and Github Copilot for assistance with the code. After using these tools, the authors reviewed and edited the content and the code as needed and take full responsibility for the content of the publication.
