# Supplementary material for: actifpTM: a refined confidence metric of AlphaFold2 predictions involving flexible regions
Source: Bioinformatics. 2025 Mar 13;41(3):btaf107. doi: 10.1093/bioinformatics/btaf107 (PMC11925850; doi:10.1093/bioinformatics/btaf107)
Supplement: btaf107_Supplementary_Data [file btaf107_supplementary_data.pdf]

# Supplementary Figures for

# actifpTM: a refined confidence metric of AlphaFold2 predictions involving flexible regions

Julia K. Varga[1], Sergey Ovchinnikov[2] and Ora Schueler-Furman[1]*

[1]Department of Microbiology and Molecular Genetics, Institute for Biomedical Research Israel-Canada, Faculty of Medicine, The Hebrew University of Jerusalem, Jerusalem 9112001, Israel
[2]Department of Biology, Massachusetts Institute of Technology, Cambridge, MA 02139
John Harvard Distinguished Science Fellowship, Harvard University, Cambridge, MA 02138

* Correspondence: ora.furman-schueler@mail.huji.ac.il

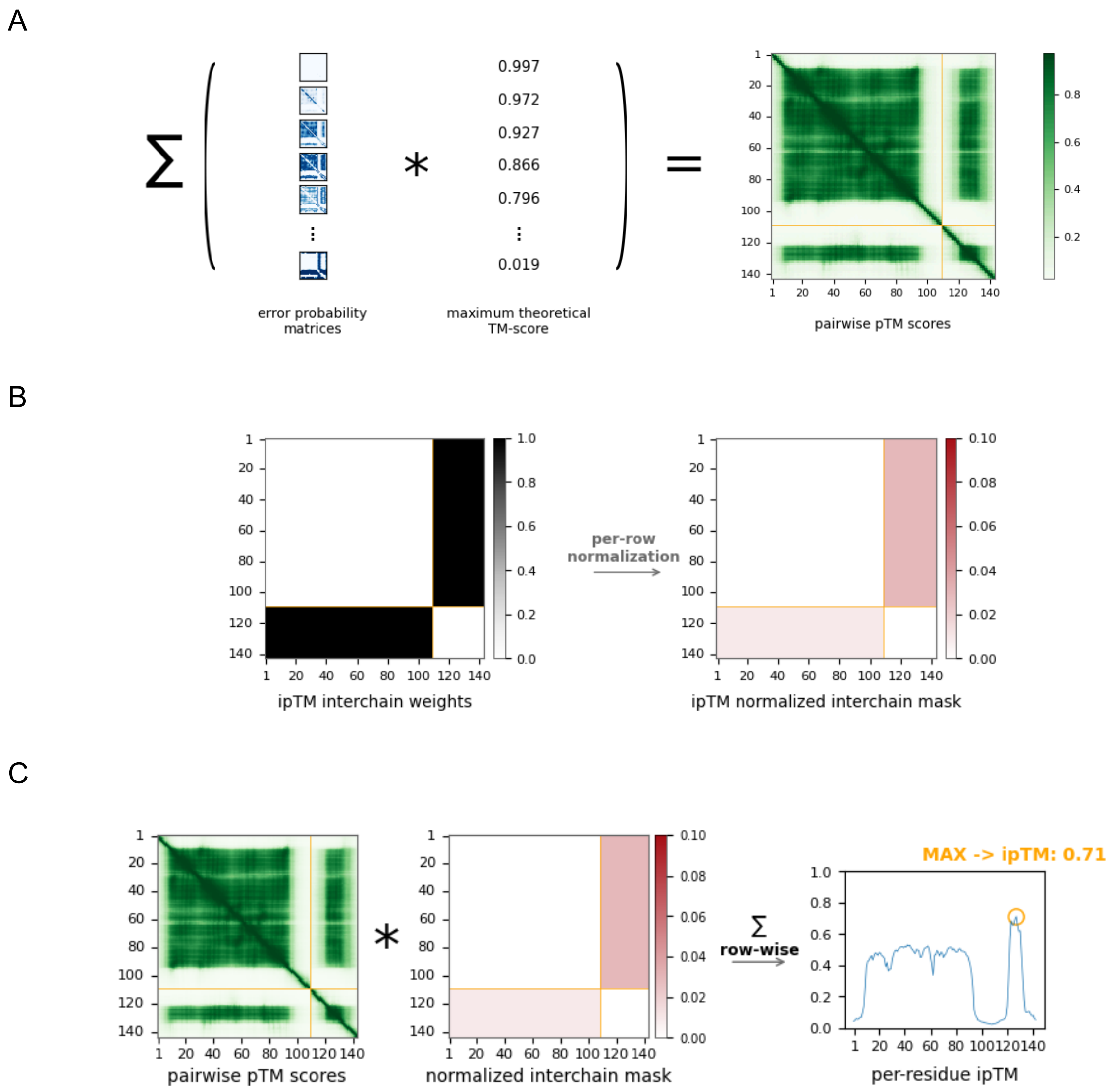


**Supplementary Figure 1. Calculation of ipTM in the AF2 pipeline. A)** A theoretical maximum TM-score is calculated from the values of the error bin centers, then pairwise matrices of each bin are multiplied by the respective theoretical maximum TM-score. Summing them for each pair of residues across bins gives rise to the pairwise TM-score matrix. **B)** The residue weights (equal weights for all residues for pTM and only interchain regions for ipTM calculation) are normalized row-wise. **C)** In the final steps of the algorithm, the pairwise pTM-matrix is multiplied by the normalized pair-residue weights. Then for each aligned residue (Y axis), the values are summarized and the maximum of these values are taken for the pTM or ipTM score.

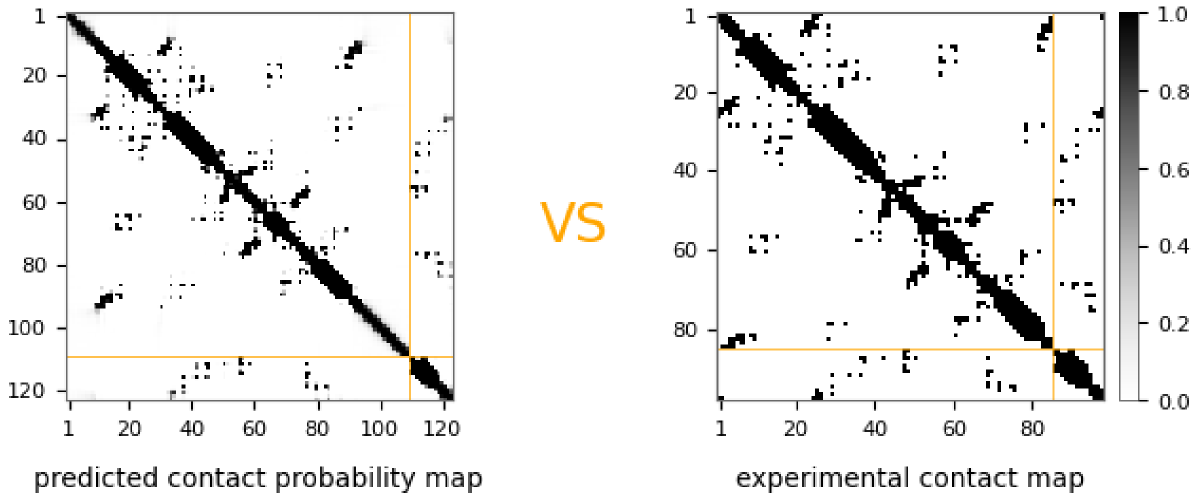


**Supplementary Figure 2. Comparison of contact probabilities and experimental contact map.** Contact probabilities derived from AF2 for a predicted shorter peptide (p53 residues 17-29, left) are in good agreement with the experimental contact map, as expected (PDB ID: 1YCR, resolved residues 17-29 in p53, right).

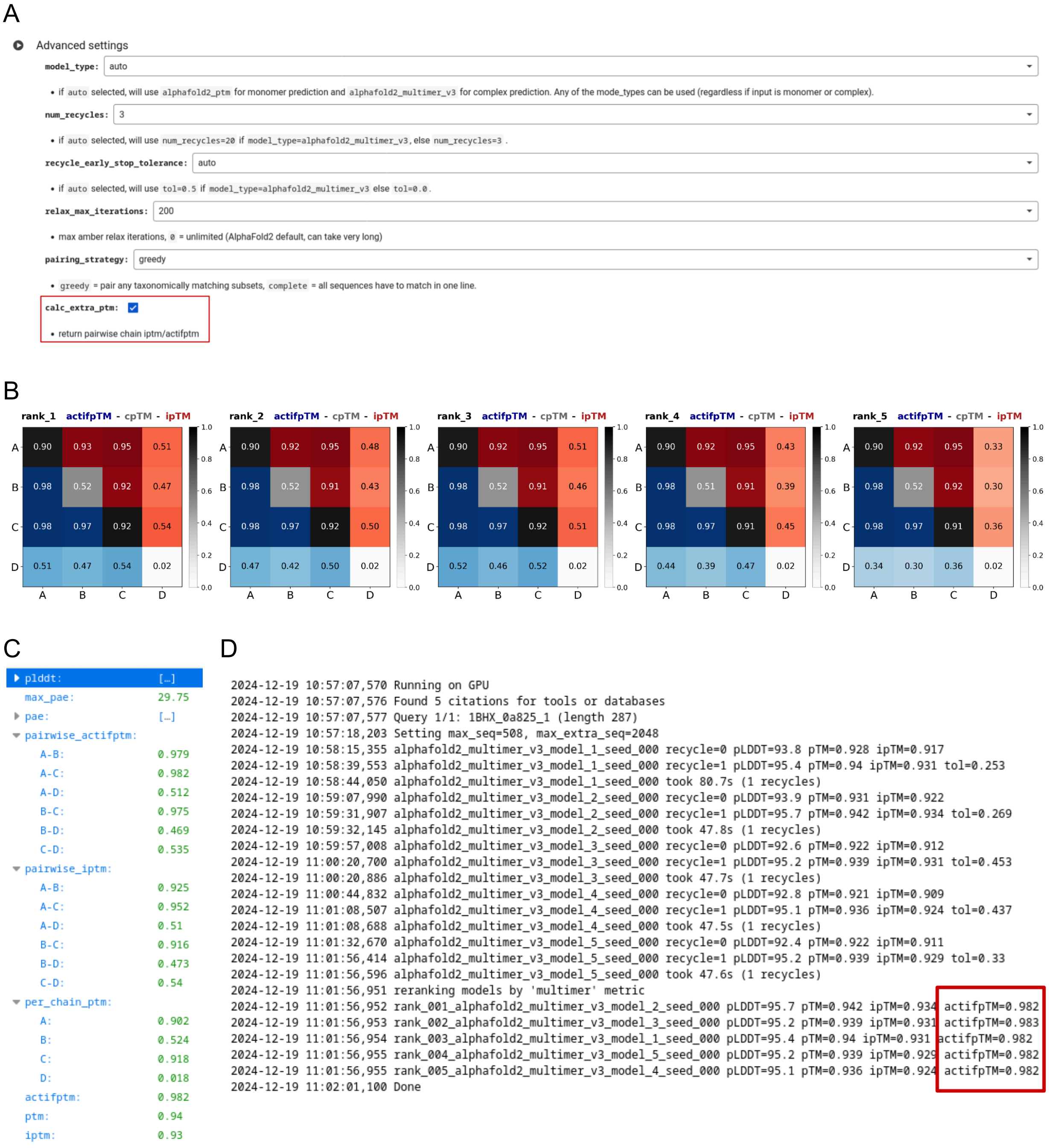


**Supplementary Figure 3. Using ColabFold to calculate actifpTM. A)** Part of the Advanced settings of https://github.com/sokrypton/ColabFold/blob/main/AlphaFold2.ipynb, that now includes the "calc_extra_ptm" option. **B-D)** Example outputs for a run, predicting the complex in PDB ID: 1BHX. **B)** A matrix of pTM values: the lower part of the triangle shows pairwise actifpTM (blue scale), the upper part shows pairwise ipTM (calculated with the standard, interchain method, red scale) and the diagonal values (grey scale) denote chain pTM-s (cpTM). **C)** Pairwise actifpTM, ipTM, chain pTM-s and actifpTM calculated for the full complex are also appended to the output json files of each model **D)** actifpTM of the full complex is also printed in the log at the end of the pipeline.
